# A demographic and epidemiological study of a Mexican chiropractic college public clinic

**DOI:** 10.1186/1746-1340-17-4

**Published:** 2009-03-19

**Authors:** Daniel A Martinez, Ronald L Rupert, Harrison T Ndetan

**Affiliations:** 1 Parker College of Chiropractic Research Institute, Parker College of Chiropractic, 2500 Walnut Hill Lane, Dallas, TX 75229, USA

## Abstract

**Background:**

Descriptive studies of chiropractic patients are not new, several have been performed in the U.S., Australia, Canada, and Europe. None have been performed in a Latin American country. The purpose of this study is to describe the patients who visited a Mexican chiropractic college public clinic with respect to demographics and clinical characteristics.

**Methods:**

This study was reviewed and approved by the IRB of Parker College of Chiropractic and the Universidad Estatal del Valle de Ecatepec (UNEVE). Five hundred patient files from the UNEVE public clinic from May 2005 to May 2007 were selected from an approximate total number of 3,700. Information was collected for demographics, chief complaints, associated complaints, and previous care sought.

**Results:**

The sample comprised 306 (61.2%) female. Most files (44.2%) were in the age range of 40–59 years (mean of 43.4 years). The most frequent complaints were lumbar pain (29.2%) and extremity pain (28.0%), most commonly the knee. Most (62.0%) described their complaints as greater than one year. Trauma (46.6%) was indicated as the initial cause. Mean VAS score was 6.26/10 with 20% rated at 8/10.

**Conclusion:**

Demographic results compared closer to studies conducted with private clinicians (females within the ages of 40–59). The primary complaint and duration was similar to previous studies (low back pain and chronic), except in this population the cause was usually initiated by trauma. The most striking features were the higher number of extremity complaints and the marked increased level of VAS score (20% rated as 8/10).

## Background

Chiropractic has become increasingly utilized worldwide. In the U.S. Chiropractic represents the third largest group of health care providers after medical doctors and dentists [1,2]. In the past, practitioners from other countries came to the U.S. for training and then returned to their homes to practice, sometimes contrary to the laws of their countries [3-8]. Presently, there are more than thirty-five chiropractic colleges and universities around the world where native populations can now train for careers in chiropractic and other alternative health care professions in their own countries [9,10]. Many of these schools are associated with outpatient teaching clinics and all utilize the local population as a patient base. Mexico is a more recent addition to this number, opening its school in August of 2001 and beginning classes with a small number of students in September of that same year. The clinic opened its doors to the public in 2005 when the beginning students started their clinical experience [11]. With two years of operation a research question can now be formulated concerning the demographics and clinical characteristics of the population seen in this clinic: Who are the people utilizing this clinic and what are they seeking care for?

Descriptive studies of chiropractic patients are not new. Recent and past studies describing patients demographics and clinical characteristics of field clinicians have been performed in the United States [12,13], Australia[14,15], Canada[16,17], and Europe [18-20]. Other studies have compared the patients at different chiropractic college teaching clinics and those of private clinicians. Sawyer and Stewart conducted a descriptive study of patients attending a chiropractic teaching clinic in Minnesota in 1981–1982 [21]. Neyiendo and others have published several descriptive and comparative studies describing patients at a single teaching clinic, describing and comparing patients at six chiropractic teaching clinics and a subsequent study using these findings to compare with patients attending private clinicians in the same area as the teaching clinics [22-25]. Walsh and Jamison in 1992 compared patients at three teaching clinics with those of three private clinics in Australia [26]. In 2003 Morschhauser et. al conducted a descriptive cross-sectional study comparing patients at on-campus clinics, off-campus clinics, and outreach clinics at four chiropractic colleges located in California, Illinois, Iowa, and Oregon [27]. Stevens performed a demographic and referral analysis of a free clinic in the Buffalo, New York area in 2007 [28]. However, there is a dearth in literature; in fact none have been identified, of related studies performed in a Latin American country. Being that Mexico is a more recent addition in this arena of health care, this would be the first description of a pure source of a Spanish speaking population.

The purpose of this study, thus, is to describe the patient population who visited the Mexican chiropractic college public clinic with respect to demographics and clinical characteristics.

## Methods

The respective Institutional Review Boards of Parker College of Chiropractic and the Universidad Estatal del Valle de Ecatepec (UNEVE) approved this study. This retrospective cross-sectional study used existing patient files for data collection. The study was conducted at the UNEVE chiropractic teaching clinic located on campus in a heavily industrial suburb northeast of Mexico City. The clinic has approximately 40 interns per year servicing the local population. Interns attend to patients for one year as part of their clinical experience.

Five hundred patient files from May 2005 to May 2007 were selected at random from an approximate total number of 3,700. Active files were stored in three 4 foot high filing cabinets, with inactive files stored in 12 cardboard boxes with approximately 200 files per box. Boxes were categorized according to year and sequence number. Files were chosen if all forms were filled out completely and all data could be ascertained. Every fifth file was to be reviewed; however, since several from each box were not filled out completely the random order was disrupted. Consecutive files from each box were reviewed until 40 qualifying files were found to reach a total of 480. Active files in cabinets up to May 2007 were similarly reviewed to fill out the study goal of 500 files. Patient's identities were protected by a unique identification code assigned to each file. This code was privy to the investigators only.

Data for each patient was abstracted from a new patient intake form. The eight-paged document included personal patient information, a systems review, a health status questionnaire, an Oswestry and Roland Morris low back questionnaire, a two-page physical exam form, and a doctor's summary report. The health status questionnaire was used by the interns to obtain most of the chief complaint characteristics such as description and onset of the complaint, quality and timing of pain complaints, Visual Analog Scale information, what actions or situations made the condition better or worse, other related symptoms, and if previous care was sought and if so what treatment was provided. Patients completed these forms themselves and/or were interviewed by the intern. The Demographic information was abstracted from the patient personal information, while the chief complaint characteristics were abstracted primarily from the health status questionnaire and doctor's summary report. A data collecting form was created to collect information on demographics (age, sex, marital status, and occupation), chief complaints (nature, duration, cause, Visual Analog Score (VAS), and associated complaints), and previous care (physician previously attended/treatment plan). See Additional file 1 and Additional file 2.

Data for this study was entered in an SPSS 15.0 (Chicago, IL) spread sheet for analysis. Simple exploratory data analysis was performed to summarize percentages of demographic and chief complaint variables, and the mean and standard deviation of duration and VAS were compared with several variables.

## Results

### Demographics

Table 1 presents the frequency of demographic variables. The sample comprised of 306 (61.2%) female. The mean (standard deviation) of the patients' age was 43.4 (15.9) years. Most (221, 44.2%) of the patients were between the ages of 40 and 59 years. About 298 (59.6%) of them were married, and 144 (28.8%) single; 134 (26.8%) performed household work while 102 (24%) performed managerial or clerical work (See Table 1).

**Table 1:** Frequency n(%) of the distribution of demographics variables

**Demographics**	**Overall**	**N(%)**
Sample	Total	500
Gender	Male	194(38.8)
	Female	306(61.2)
Age	<20 Yrs	39(7.8)
	20–39 Yrs	161(32.2)
	40–59 Yrs	221(44.2)
	>59 Yrs	79(15.8)
Marital Status	Married	298 (59.6)
	Single	144(28.8)
	Divorced	11(2.2)
	Widowed	30(6.0)
	Civil Union	17(3.4)
Occupation	Housework	134 (26.8)
	Blue collar/manual	66(13.2)
	Professional/office	120(24.0)
	Student	57(11.4)
	Shopkeeper	74(14.8)
	Other	49(9.8)

### Chief complaint

Table 2 presents frequency of the chief complaint variables. A secondary and tertiary complaint was included if the patient reported more than one area of complaint. Lumbar (29.2%) and extremities (28.0%) were the most prevalent primary complaints. Secondary complaints were lumbar (77/281, 27.4%) and extremities (48/281, 17.1%). Sacrum and coccyx (16/76, 21.1%), and extremities (19/76, 25.0%) were tertiary complaints. In total, extremity complaints were recorded in 207 of the files (41.4%), with 101 (48.8%) of these listed as knee complaints.

**Table 2:** Frequency n(%) of the chief complaint variables

**Primary Complaint**	**N(%)**	
Head and Neck	82(16.4)	
Thoracic	63(12.6)	
Lumbar	146(29.2)	
Pelvis	42(8.4)	
Sacrum and coccyx	19(3.8)	
Extremities	140(28.0)	
Radiculopathy	2(.4)	
Other	6(1.2)	
Total	500(100)	

**Secondary Complaint**		
Head and Neck	15(5.3)	
Thoracic	27(9.6)	
Lumbar	77(27.4)	
Pelvis	33(11.7)	
Sacrum and coccyx	41(14.6)	
Extremities	48(17.1)	
Radiculopathy	35(12.5)	
Other	5(1.8)	
Total	281/500(56.2)	

**Tertiary Complaint**		
Head and Neck	2(2.6)	
Thoracic	3(3.9)	
Lumbar	13(17.1)	
Pelvis	9(11.8)	
Sacrum and coccyx	16(21.1)	
Extremities	19(25.0)	
Radiculopathy	13(17.1)	
Other	1(1.3)	
Total	76/500(15.2)	

Extremity complaints*	**Total N(%)**	**≥90 days N(%)**
Shoulder	50(24.2)	33(22.4)
Elbow	9(4.3)	4(2.7)
Hand	11(5.3)	8(5.4)
Hip	8(3.9)	4(2.7)
Knee	101(48.8)	82(55.8)
Ankle	28(13.5)	16(10.9)
Total	207/500 (41.4)	147/500 (29.0)

**Duration of complaint**	**N(%)**	
Acute (<90 days)	122(24.4)	
Chronic (> = 90 days)		
< 365 days	68(13.6)	
365 days +	310(62)	
Total	500(100)	

**Cause**		
Trauma	235(47.0)	
Work Related	74(14.8)	
Sports Related	68(13.6)	
Idiopathic	98(19.6)	
Other	25(5.0)	
Total	500(100)	

**Visual Analog Scale**		
0	1(.2)	
1	2(.4)	
2	12(2.4)	
3	31(6.2)	
4	52(10.4)	
5	97(19.4)	
6	69(13.8)	
7	70(14.0)	
8	100(20)	
9	48(9.6)	
10	18(3.6)	
Total	500(100)	

**Related Symptoms**		
None	233(46.6)	
Fever	7(1.4)	
Night sweats	48(9.6)	
Dizziness	87(17.4)	
Blurred vision	64(12.8)	
Fatigue	144(28.8)	
Other	61(1.22)	
Total	500(100)	

**Previous Attended**		
None	216(43.2)	
Medical	162(32.4)	
Physiotherapist	39(7.8)	
Alternative health care	59(11.8)	
Folk medicine	46(9.2)	
Other	3(.6)	
Total	500(100)	

**Treatment**		
None	215(43.0)	
Drug therapy	126(25.2)	
Surgery	2(.4)	
Physiotherapy	52(10.6)	
Other	150(30.0)	
Total	500(100)	

* Includes primary, secondary, and tertiary complaints

Duration was calculated in days with a range of 1 day to 10,950 days (30 years). Chronic (75.6%) was assigned to conditions greater than 90 days duration [29], with most (62%) of these describing their complaints as greater than one year. The mean duration of the chief complaint was 1,494.92 days (4.10 years), with the 40 to 59 age range representing the highest number 178/500 (36%) of chronic cases (Table 3). Trauma in 47.0% of cases was indicated as the initial cause. The duration for causes due to trauma was 1,603.49 days (6.32 years). The most common description of trauma was a slip and fall either to the posterior or to the knees. Of the 207 extremity complaints 147 (29.4%) were greater than three months duration with 55.8% (82/147) of these classified as knee complaints. The most frequent primary complaint (lumbar) averaged 2,016.61 days (5.52 years) duration, while the second most frequent complaint (extremities) averaged 976.08 days (2.67 years), with knee complaints averaging 1,633.68 days (4.48 years). The mean of the VAS was 6.26/10 (SD 2.0), with 20% classifying their pain as 8/10. Mean VAS scores were above 6/10 in all categories above 20 years of age and above 5.5/10 in the under 20 year's age group (Table 4). The mean VAS score when trauma was listed as the cause was 6.4/10 (1.96). Of the two most prevalent primary complaints, lumbar conditions VAS scores were listed as 6.4/10 (1.98) and extremities as 6.2/10 (2.14) with knee complaints 6.6/10 (2.23). "No previous attention" was listed in 43.2% of cases; however, 32.4% sought "Medical attention" first. Of these, drug therapy was sought in 25.2% of cases. The corresponding mean duration and VAS scores were1,225.77 days (3.36 years) and 5.8/10 (SD 1.99) for "No previous attention" and 1,857.65 days (5.09 years) and 6.8/10 (SD1.94) for "Medical attention" (Tables 5 and 6). About 46.6% did not list a related symptom.

**Table 3:** Mean of duration with chief complaint

**Chief Complaint**	**Mean Days(years)**	**Standard deviation Days(years)**	**N**
Head and Neck	1561.99(4.28)	1875.67(5.14)	82
Thoracic	1435.11(3.39)	1679.27(4.60)	63
Lumbar	2016.61(5.52)	2643.98(7.24)	146
Pelvis	1453.57(3.98)	2308.61(6.3)	42
Sacrum and coccyx	1592.95(4.36)	2131.57(5.34)	19
Extremities	976.08(2.68)	1697.50(4.65)	140
Radiculopathy	165.00(0.45)	190.92(0.52)	2
Other	1040.83(2.85)	897.30(2.46)	6
Total	1494.92(4.10)	2128.81(5.83)	500

**Table 4:** Mean of visual analog scale with category of age

**Age Range**	**Mean**	**Standard deviation**	**N**
< 20 years	5.54	1.833	39
20–39 years	6.31	1.966	161
40–59 years	6.20	2.031	221
> 60 years	6.65	2.063	79
Total	6.26	2.012	500

**Table 5:** Mean of duration with type of previous care

**Type of previous care**	**Mean Days(years)**	**Standard deviation Days(years)**	**N**
None	1225.77(3.36)	1827.56(5.00)	216
Medical	1857.65(5.09)	2527.96(6.93)	160
Physiotherapy	1378.29(3.78)	1962.84(5.38)	31
Alternative	1842.44(5.05)	2123.65(5.82)	50
Folk Medicine	1236.68(3.39)	1900.58(5.21)	40
Other	385.00(1.06)	335.45(0.92)	3
Total	1494.92(4.10)	2128.81(5.83)	500

**Table 6:** Mean of visual analog scale with type of previous care

**Type of previous care**			
None	5.80	1.999	216
Medical	6.83	1.940	160
Physiotherapy	6.48	2.365	31
Alternative	6.24	1.611	50
Folk Medicine	6.35	2.032	40
Other	5.33	1.155	3

## Discussion

There was a predominance of women (61.2%) in this study, which is consistent with studies performed in other countries [12-28]. However, this proportion is greater than the proportion in the general population of the valley of México which is 51.6% female [30]. The most dominant age group of 40–59 years is consistent with prior descriptive studies of patients seen by field doctors [12-20], but this group is older than the dominant group in studies comparing patients at teaching clinics [21-28]. It is, however, consistent with the dominant age group of patients seen in the teaching clinics where the population was predominantly Hispanic [22,27].

The most frequent primary complaint of low back pain (29.2%) compares with numerous other studies, however, the second most frequent primary complaint of extremity pain (28.0%), most commonly the knee, is considerably higher. This is nearly 2–4 times higher than field studies performed in the United States [12,13], and Europe [18-20], and almost twice that of Australian studies [15,26]. Nyiendo's study of six colleges in 1989 provides a closer comparison with a range of 17–22% among the participating clinics [22]. The percentage of extremity complaints is even higher when the secondary and tertiary complaints are included (41.4%). This would seem to coincide with the high percentage of causes due to trauma (47.0%). All age groups reported more traumas than any other cause in their respective groups.

It can be said that this population suffers from chronic complaints. By far, the majority of patients had complaints greater than three months, with a high percentage of these greater than one year (Table 2). Other studies have recorded similar chronic conditions [19,22,27], but this population averaged over four years with their complaints (Table 3). This may suggest that either patients have chosen to live or have had to live with their conditions or had sought other care without adequate results before seeking chiropractic care. It is also possible, due to the paucity of chiropractic clinics throughout the valley of México that chiropractic care has just not been an available option until recently.

According to Mersky, pain is defined as "an unpleasant sensory and emotional experience" that is "always subjective." [31]. Pain perceptions rated high with this population. Twenty percent of the total study population defined their pain as an 8/10 on the VAS scale. Multiple comparisons among age groups only showed a significant difference between the <20 and >60 years age groups, even then the <20 group listed a mean of greater than 5/10 on the VAS (Table 4). Pain perception was still high even after initial care; for example, mean VAS scores after medical treatment was 6.8/10 (1.940) in 32% of cases (Table 6). In accordance with a study performed by Nyiendo comparing patients at teaching colleges with field clinicians, this compares more closely to the field clinicians than with the teaching clinics [22]. This study was limited to initial VAS scores and subsequent scores are not available for comparison. Associated symptoms were listed on the intake form for the patient to choose from. Despite 46.6% listing no other symptoms, it was not always clear if the patient who did list a symptom understood that it must relate to the primary cause.

Even though the highest percentage of files listed no previous treatment, the question on the intake form could be misleading. The question was stated as "Have you been attended to previously for these health problems?" (¿Ha sido atendido previamente por estos problemas de salud?). It was not clear whether the patient attended their problem immediately or if the patient treated themselves. Also, there was some confusion with this question concerning alternative health care providers with some of the folk medicine providers. Mexico has a strong tradition of folk healers, one being a "huesero" (bonesetter) [32], who are sometimes confused with chiropractors by the general public and it was not always clear who was being indicated.

Certain limitations of this study are acknowledged. This study was purely descriptive in nature and limited to patient recall and responses or the interpretation of the response by the intern and any attempt to verify the patient response with clinical findings was limited by the experience of the intern; therefore, the diagnostic categories used were very general. The randomization was weakened due to the inconsistency of completeness of the files; however, randomization may have been achieved if the files were first excluded by the stated criteria and then randomly selected. Some questions in the intake forms made some categorization of previous care difficult. While the sample size may have been representative of the clinic, it may not be representative of all of Mexico.

Despite these limitations, it is possible to gain an insight as to the patient population that attended the clinic. This study provides a demographic and epidemiological foundation for future investigation; however, recommendations for strengthening intake forms and policies of the clinic may help to facilitate future desired data collection. Practical applications of this study would allow practitioners to better understand and prepare for the most common conditions that may present in the clinic.

## Conclusion

This study describes the profile of patients who visited a Mexican chiropractic college public clinic with respect to demographics and clinical characteristics. Demographic results of mostly females within the ages of 40–59 are more consistent with studies conducted with private clinicians rather than with studies conducted with teaching clinics. The primary complaint and duration is similar to both field and teaching clinic studies (low back pain and chronic), except in this population the cause was usually initiated by trauma (specifically falls). The most striking features, however, were the higher number of extremities complaint and the marked increased level of VAS score (20% rated as 8/10). Future studies may explore the treatment and prevention of knee complaints.

## Competing interests

The authors declare that they have no competing interests.

## Authors' contributions

DM contributed to the design, carried out the data collection, performed the literature search, and drafted and wrote the manuscript. RR contributed to the supervision, concept and design, and editing and revision for the intellectual content of the article. HN provided statistical analysis of the data, and critical review of the manuscript. All authors read and approved the final manuscript.

## Supplementary Material

Additional file 1 **Data collecting form in Spanish. **The collecting form was dived into category, where the information could be found in the patient file, and space to record the information. Click here for file

Additional file 2 **English translation of collecting form. **English translation of the collecting form (Additional file 1). Click here for file
