# Supplementary material for: A demographic and epidemiological study of a Mexican chiropractic college public clinic
Source: Chiropr Osteopat. 2009 Mar 19;17:4. doi: 10.1186/1746-1340-17-4 (PMC2667450; doi:10.1186/1746-1340-17-4)
Supplement: Additional file 1 — Data collecting form in Spanish. The collecting form was dived into category, where the information could be found in the patient file, and space to record the information. [file 1746-1340-17-4-S1.doc]

**Datos De Universidad Estatal del Valle de Ecatapec Expediente**

**Demográfica**

Categoría Localización en la forma Información

| 1. Edad | Personales no Patológicos: *Fecha de nacimiento* |  |
| --- | --- | --- |
| 2. Sexo | Historia Clínica | M ____ F ____ ­­­ |
| 3. Estado Civil | Personales no Patológicos | C ____ S____ D____ V____ U____ |
| 4 Ocupación | Personales no Patológicos |  |

**Padecimiento actual**

**A. Características generales**

Categoría Localización en la forma Información

| 5. Naturaleza de Dolor | Cuestionario actual de estado de salud: *Por favor describa en forma breve sus problemas…* |  |
| --- | --- | --- |
| 6. Duración | Cuestionario actual de estado de salud: ¿*Como y cuando comenzó este problema?* |  |
| 7. Resultado VAS | Cuestionario actual de estado de salud: *De acuerdo con la intensidad, encierren…* |  |
| 8. Causa | Cuestionario actual de estado de salud: *¿Como y cuando comenzó este problema?* |  |
| 9. Otros Síntomas | Cuestionario actual de estado de salud: *¿Ha tenido algunos otros síntomas?* |  |

**B. Cuidado previo**

Categoría Localización en la forma Información

| 10. Clase de Asistencia Sanitaria | Cuestionario actual de estado de salud: *¿Ha sido atendido previamente por estos problemas de salud?* |  |
| --- | --- | --- |
| 11. Tratamiento | Cuestionario actual de estado de salud: *Si es así, por favor describa en qué consistieron los tratamientos.* |  |
