# Supplementary material for: A demographic and epidemiological study of a Mexican chiropractic college public clinic
Source: Chiropr Osteopat. 2009 Mar 19;17:4. doi: 10.1186/1746-1340-17-4 (PMC2667450; doi:10.1186/1746-1340-17-4)
Supplement: Additional file 2 — English translation of collecting form. English translation of the collecting form (Additional file 1). [file 1746-1340-17-4-S2.doc]

**Data From the Universidad Estatal del Valle de Ecatapec forms**

**Demographics**

Category Location in the form Data

| 1. Age | Personal information : *Date of Birth* |  |
| --- | --- | --- |
| 2. Sex | Clinical History | M ____ F ____ ­­­ |
| 3. Marital Status | Personal information | M ____ S____ D____ W____ C____ |
| 4 Occupation | Personal information |  |

**Chief complaint**

**A. General Characteristics**

Category Location in the form Data

| 5. Nature of pain | Health status questionnaire: *Please, briefly describe your problem* |  |
| --- | --- | --- |
| 6. Duration | Health status questionnaire: *How and when did this problem start?* |  |
| 7. VAS score | Health status questionnaire: *Rate your pain according to the scale below* |  |
| 8. Cause | Health status questionnaire: *How and when did this problem start?* |  |
| 9. Other Health Complaints | Health status questionnaire: *Have you had any other symptoms?* |  |

**B. Previous care**

Category Location in form Data

| 10. Type of Physician | Health status questionnaire: *Have you been attended to previously for this/ these health problems?* |  |
| --- | --- | --- |
| 11. Treatment | Health status questionnaire: *If yes, please describe your treatment* |  |
